# Clinicopathologic study of mantle cell lymphoma with epstein-barr virus infection: A case series and literature review

**DOI:** 10.3389/fonc.2022.933964

**Published:** 2022-08-02

**Authors:** Xiaoju Li, Fanlin Zhou, Shijie Li, Yangyang Wang, Jianing Fan, Xiao Liang, Yan Peng, Yudi Jin, Weiyang Jiang, Fang Liu, Yixing Zhou, Shuke Liu, Tao Wang, Yi Peng, Jianbo Xiong, Jia Liu, Jing Zhang, Changqing He, Hui Zhang, Yu Li

**Affiliations:** ^1^ Department of Pathology, Chongqing University Cancer Hospital, Chongqing, China; ^2^ Department of Pathology, School of Basic Medicine, Chongqing Medical University, Chongqing, China; ^3^ Bioengineering College, Chongqing University, Chongqing, China; ^4^ School of Medicine, Chongqing University, Chongqing, China; ^5^ Institute of Neuroscience, School of Basic Medicine, Chongqing Medical University, Chongqing, China; ^6^ Department of Hematology-Oncology, Chongqing Key Laboratory of Translational Research for Cancer Metastasis and Individualized Treatment, Chongqing University Cancer Hospital, Chongqing, China

**Keywords:** Epstein–Barr virus, mantle cell lymphoma, non-neoplastic bystander cells, background cells, clinicopathological features

## Abstract

**Background:**

Mantle cell lymphoma (MCL) with Epstein–Barr virus (EBV) infection is rarely reported. The objective of this study was to analyze the prevalence and clinicopathological features of MCL with EBV infection in the largest series thus far.

**Methods:**

After screening 138 cases of MCL, we identified eight cases of MCL with EBV infection.

**Results:**

Most of them (7/8) had non-neoplastic bystander cells with positivity for EBV and no expression of latent membrane protein 1 (LMP1) and EBV nuclear antigen 2 (EBNA2). The cases of MCL with EBER positivity did not have abnormal immune function or other lymphomas. Moreover, their histopathological morphology was indicative of classical MCL. Cases of MCL with EBER positivity exhibited statistically significant differences in lactate dehydrogenase, anemia status, and MCL international prognostic index grouping (P=0.008, P=0.02, P=0.001, and P=0.011, respectively). The differences between the two groups in age, sex ratio, clinical manifestations, and immunohistochemical phenotypes were not statistically significant.

**Conclusions:**

The incidence of MCL with EBV infection was low (5.8%). Clinicopathologically, cases of MCL with EBER positivity were similar to their EBV-negative counterparts. Our findings revealed that most cells infected by EBV in MCL are background cells rather than tumor cells. This is inconsistent with data from previous studies, indicating that tumor cells in MCL may not be prone to EBV infection.

## Introduction

Epstein–Barr virus (EBV), also termed human herpes virus 4, exhibits an infection rate >90% worldwide (1). Etiologically, EBV is linked to lymphoproliferative lesions and malignant lymphomas of B-, T-, and natural killer-cell origin, manifesting in the form of asymptomatic latent infection in the vast majority of individuals (2, 3). Some EBV infections are events that persist in the B lymphoid system, while others are the result of viral entry into T cells or natural killer cells (3). EBV infection is associated with disease progression, and patients with EBV-positive lymphoma, diffuse large B-cell lymphoma, have a poor prognosis (4). Recent studies have found that some mature small B-cell lymphomas, such as follicular lymphoma (FL) and marginal zone lymphoma (MZL), are associated with EBV infection (5–8). Most of these cases are linked to aging, immunodeficiency, immunosuppression, and transplantation.

At present, there are few studies on EBV-associated mantle cell lymphoma (MCL), and most of them are case reports (9–16). These studies have established the connection between EBV and MCL. However, due to the complex life cycle and biological behavior of EBV, its role in MCL remains unknown. According to the currently available research studies, it is hypothesized that EBV may be associated with the transformation of MCL to Hodgkin’s lymphoma (HL) or more aggressive lymphoma (16). Therefore, it is necessary to correctly recognize and diagnose MCL with EBV infection.

In this study, we analyzed eight MCL cases with EBV infection, including their clinical manifestations and laboratory findings, to investigate whether MCL with EBV infection has unique clinicopathological features.

## Materials and methods

### Patient selection

A total of 138 cases of MCL in the Department of Pathology at Chongqing Medical University and Chongqing University Cancer Hospital(China) from January 2014 to November 2019 were retrospectively examined. The diagnosis was confirmed by two chief hematopathologists. A diagnostic criterion was established according to the 4th revised edition of the World Health Organization classification of hematopoietic tumors and lymphoid tissues. This research was approved by the Research Ethics Committee of the Chongqing University Cancer Hospital.

### Clinical and pathological characteristics assessment

Clinical data, including age, sex, biopsy site, systemic lymphadenopathy, extra-lymph node lesions, bone marrow involvement, B symptoms, Ann Arbor stage, and MCL international prognostic index (MIPI) groups, were analyzed. Additionally, laboratory examinations, including red blood cell (RBC) count, hemoglobin count, lactate dehydrogenase (LDH), leukocyte count, as well as the absolute value of monocytes and beta-2-microglobulin (β2-MG), were analyzed.

Slides were reviewed, including biopsy of the lymph node or mass and bone marrow biopsies in selected cases. The immunohistochemical indices (CD20, CD79a, CD5, cyclin D1 [CCND1], SRY-box transcription factor 11 [SOX11], CD43, CD10, B-cell lymphoma 6 [BCL-6], BCL-2, Ki-67 index, Latent membrane protein 1 (LMP1) and EBV nuclear antigen 2 (EBNA2)), as well as data on the molecular detection of the CCND/immunoglobulin heavy chain (IgH)-t (11, 14) of patients were retrospectively collected. Antibodies of CD20, CD79a, CD5, cyclin D1, SOX11, CD43, CD10, BCL-6, BCL-2, Ki-67 were purchased from Maixin Biotechnology Co., Ltd. (Fuzhou, China). Antibodies of LMP1 and EBNA2 were purchased from Zhongshanjinqiao Biotechnology Co., Ltd. (Beijing, China).

### In situ hybridization (ISH) for EBV and CD79a/CD34 immunohistochemical double labeling

EBV detection was performed by ISH using EBV-encoded RNA (EBER) probes (Zhongshanjinqiao Biotechnology Co., Ltd.) (Beijing, China) according to the experimental protocol provided by the manufacturer. This technique can determine the type of cells infected by EBV. The positive signal of EBER was located in the nucleus and was shown with a brownish yellow/red. The positive signal of CD79a/CD3 was located on the cell membrane of B/T lymphocytes. Therefore, only tumor cells that exhibit positivity both in the nucleus (EBER) and membrane (CD79a) are judged to be EBV-positive tumor cells. Subsequently, specimens were observed using a microscope (Olympus, Japan).

### Statistical analysis

Data are presented as the mean ± standard deviation, range, frequencies (number of cases), and percentages as appropriate. Student’s t-test using two independent samples was employed for comparisons between groups; the median and quartile interval were used to determine data skewness, and the Mann–Whitney U test was used for comparison between groups. Statistical analyses were performed using the SPSS software (version 17.0; IBM Corp, Armonk, NY, USA). P-values < 0.05 denoted statistically significant differences.

## Results

### Clinical features of EBV-positive and EBV-negative MCL patients

Screening of 138 cases of MCL revealed eight EBER-positive cases. Therefore, the infection rates with and without EBV in MCL were 5.8% (8/138) and 94.2% (130/138), respectively. Due to the lack of complete clinical data and pathological tissue, the data of 52 patients were excluded. Therefore, 8 EBER positive MCL patients and 78 EBER negative MCL patients were included. Clinical characteristics of MCL patients with and without EBER positivity were analyzed and compared using the chi-squared test (**Table 1**). There were statistically significant differences in RBC count, hemoglobin count, LDH, and MIPI groups (P=0.008, P=0.02, P=0.001, and P=0.011, respectively). RBC and LDH increased in all MCL patients with EBER positivity, and the MIPI score indicated medium/high risk. There were no significant differences between MCL patients with and without EBER positivity for the mean age (P=0.342), age distribution (P=0.46), sex (P>0.999), systemic lymphadenopathy (P=0.343), extra-lymph node lesions (P=0.147), bone marrow involvement (P=0.195), B symptoms (P=0.588), Ann Arbor stage (P=0.585), and leukocyte count (P=0.558), as well as absolute value of monocytes (P=0.611) and β2-MG (P>0.999). Of the eight MCL patients with EBER positivity, six were males (75%) and two were females (25%), showing a slight male predominance (male:female=3:1). The median age was 66.5 years (range: 43–80 years); two patients were aged <60 years (25%); and six cases were aged ≥60 years (65%).

**Table 1:** Comparison of clinical characteristics between patients with and without EBER positivity.

Clinical features	MCL with EBER positivity (%) (n=8)	MCL without EBER positivity (%) (n=78)	*P*
RBC normal abnormal	0 (0.00) 8 (100.00)	38 (48.72) 40 (51.28)	0.008
Hemoglobin Count normal abnormal	0 (0.00) 8 (100.00)	34 (43.59) 44 (56.41)	0.02
LDH normal up	0 (0.00) 6 (100.00)	54 (72.00) 21 (28.00)	0.001
MIPI staging Low medium high	0 (0.00) 4 (66.67) 2 (33.33)	39 (52.00) 31 (41.33) 5 (6.67)	0.011

P<0.05, the difference is statistically significant.

All patients had multiple lymph node enlargement; seven patients (87.5%) had extra-lymph node lesions, whereas one patient (12.5%) did not have extra-lymph node lesions. Four patients (50%) had bone marrow involvement, two patients (25%) did not exhibit bone marrow involvement, and bone marrow biopsy was not performed in two cases (25%). Hepatosplenomegaly was found in three cases (37.5%); the remaining five cases (62.5%) did not have hepatosplenomegaly. All patients had decreased RBC and hemoglobin counts; two cases (25%) had increased absolute value of monocytes; six cases (75%) had normal absolute value of monocytes; and one (12.5%) and seven patients (87.5%) had increased and normal white blood cell counts, respectively. Serum LDH levels were elevated in six cases (75%), and could not be evaluated in two cases (25%). β2-MG was elevated in three patients (37.5%), normal in one patient (12.5%), and not detected in four patients (50%). The patients did not show immunosuppression/immunodeficiency or B symptoms. Two patients (25%) and four patients (50%) had Ann Arbor stages IIIA and IVA, respectively. The Ann Arbor stage could not be clearly assessed in two patients (25%) without bone marrow biopsy. According to the MIPI score, four (50%) and two (25%) patients were in the medium- and high-risk groups, respectively. It was not possible to detect LDH in two patients (25%); thus, these patients were not evaluated for MIPI. One patient was treated with CHOP (cyclophosphamide, adriamycin, vincristine, and prednisone), three patients were treated with E-CHOP (CHOP and etoposide), and one patient was treated with CHOE (cyclophosphamide, adriamycin, vincristine, and etoposide); three patients were not treated with chemotherapy (**Table 2**).

**Table 2:** Clinicopathologic features of MCL with EBER positivity.

	Patient 1	Patient 2	Patient 3	Patient 4	Patient 5	Patient 6	Patient 7	Patient 8
**Age** **(years)**	**80**	**64**	**66**	**43**	**68**	**59**	**74**	**67**
**Sex**	**F**	**M**	**M**	**M**	**M**	**F**	**M**	**M**
**Multiple lymph nodes**	**Yes**	**Yes**	**Yes**	**Yes**	**Yes**	**Yes**	**Yes**	**Yes**
**Extranodal lesion**	**Yes**	**Yes**	**Yes**	**Yes**	**No**	**Yes**	**Yes**	**Yes**
**immune functions**	**No**	**No**	**No**	**No**	**No**	**No**	**No**	**No**
**BM** **involvement**	**ND**	**Yes**	**ND**	**Yes**	**Yes**	**Yes**	**No**	**No**
**Hepatosplenomegaly**	**No**	**Yes**	**No**	**Yes**	**Yes**	**No**	**No**	**No**
**B symptom**	**No**	**No**	**No**	**No**	**No**	**No**	**No**	**No**
**Ann Arbor** **Staging**	**NA**	**IVA**	**NA**	**IVA**	**IVA**	**IVA**	**IIIA**	**IIIA**
**WBC**	**N**	**N**	**N**	**Up**	**N**	**N**	**N**	**N**
**RBC/Hb**	**Down**	**Down**	**Down**	**Down**	**Down**	**Down**	**Down**	**Down**
**LDH**	**Up**	**Up**	**ND**	**Up**	**ND**	**Up**	**Up**	**Up**
**β2** **Microglobulin**	**ND**	**ND**	**ND**	**ND**	**Up**	**N**	**Up**	**Up**
**MIPI**	**MRG**	**HRG**	**NA**	**HRG**	**NA**	**MRG**	**MRG**	**MRG**
**Treatment**	**Conservative treatment**	**Give up**	**Conservative treatment**	**E-CHOP**	**E-CHOP**	**CHOP**	**CHOE**	**E-CHOP**
**OS** **(month)**	**6**	**NA**	**13**	**NA**	**60**	**NA**	**13**	**NA**

ND, Not Done; N, Normal; MRG, Medium risk group; HRG, High risk group; NA, Not acquired.

### Histological and immunophenotypic analysis of EBV-positive and EBV-negative MCL patients

The chi-squared test was used to analyze and compare the immunohistochemical findings of MCL patients with and without EBER positivity (**Table 3**). There was no significant difference in the expression of CD5 (P=0.641), SOX11 (P=0.17), CD10 (P>0.999), BCL-6 (P=0.396), and Ki-67 (P=0.707), with 30% as the threshold between MCL patients with and without EBV infection. However, the difference in CCND1 expression between the two groups was statistically significant (P<0.001). CCND1 expression was negative in part of MCL patients without EBV infection (5/78), but positive in MCL patients with EBV infection.

**Table 3:** Immunohistochemical comparison of patients with and without EBER positivity.

IHC	MCL with EBV infection (%) (n=8)	MCL without EBV infection (%) (n=78)	*P*
CD5 + -	6 (75.00) 2 (25.00)	63 (81.82) 14 (18.18)	0.641
SOX11 + -	3 (37.50) 5 (62.50)	46 (83.64) 9 (16.36)	0.17
BCL-6 + -	3 (37.50) 5 (62.50)	17 (22.97) 57 (77.03)	0.396
Ki-67 (%) ≤30 >30	4 (50.00) 4 (50.00)	48 (61.54) 30 (38.46)	0.707

**Figure 1:**
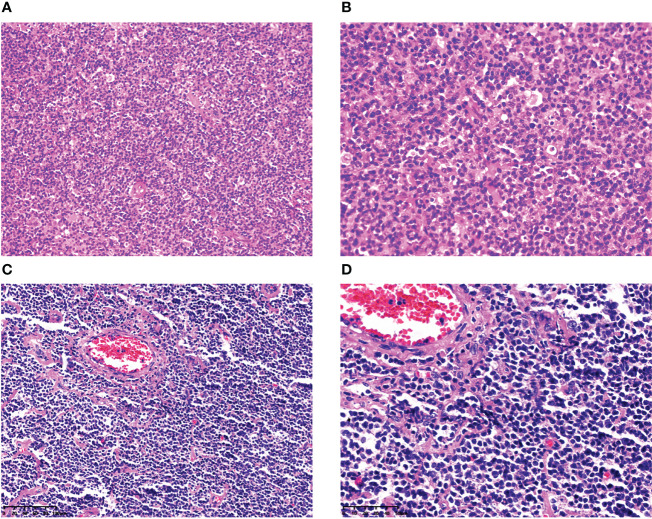
Histopathological features of MCL with EBER positivity. In case 1, at low magnification (×200), the tumor cells were small-to-medium-sized lymphocytes, and the cell morphology was single **(A)**. At high magnification (×400), the tumor nucleus was slightly irregular, similar to the central cells, the nucleolus was not obvious, mitosis was rare, and tissue-like cells were observed in the tumor background; however, HRS-like cells were not found **(B)**. In case 4, at low magnification (×200), the tumor cells were small-to medium-sized lymphocytes, with scattered vitreous degeneration of blood vessels **(C)**. At high magnification (×400), the nucleus of the tumor was slightly irregular, similar to the central cells, the nucleolus was not obvious, mitosis was rare, and there were no HRS-like cells in the background **(D)**.

In the eight MCL patients with EBV infection, the morphology was indicative of classical MCL, the tumor cells were small-to-medium-sized lymphocytes, the cell morphology was single, the nucleus was slightly irregular, similar to the central cell, the nucleolus was not obvious, mitosis was rare, scattered vascular vitreous degeneration was observed, and tissue-like cells were observed in the tumor background; however, Hodgkin and Reed/Sternberg (HRS)-like cells were not observed (**Figure 1**, cases 1 and 4).

**Figure 2:**
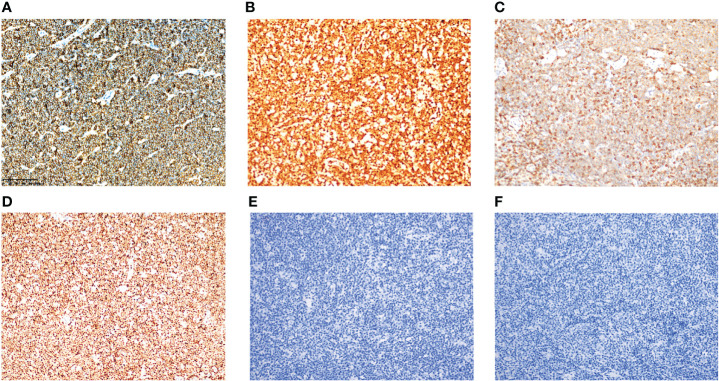
Immunohistochemical expression of MCL with EBER positivity. Tumor cells were positive for CD79a **(A)** (case 2), CCND1 **(B)** (case 2), CD5 **(C)** (case 4), and SOX11 **(D)** (case 3). Tumor cells were negative for LMP1 **(E)** (case 1) and EBNA2 **(F)** (case 1). All images were captured at ×200 magnification.

CD20, CD79a, and CCND1 were diffusely expressed in all patients (100%) (**Figures 2A, B**, case 2). Six cases (75%) expressed CD5 (**Figure 2C**, case 4), whereas two cases (25%) did not express. Three cases (37.5%) expressed SOX11 (**Figure 2D**, case 3), whereas five cases (62.5%) did not. Seven cases (87.5%) expressed BCL-2, whereas one case (12.5%) did not. Three cases (37.5%) expressed BCL-6, whereas five cases (62.5%) did not. LMP1 (**Figure 2E**, case 1) and EBNA2 (**Figure 2F**, case 1) were not detected in any cases. Ki-67 ranged 5–80%; four cases (50%) were ≤30% and four cases (50%) were >30%. The results of immunohistochemistry and CCND/IgH-t (11;14) chromosome rearrangement and translocation in tissues of these eight cases are shown in **Table 4**.

**Table 4:** Immunophenotype and molecular phenotype of MCL with EBER positivity.

Case	CD20	CD79α	CD5	CyclinD1	SOX11	CD43	CD10	BCL-6	BCL-2	LMP-1	EBNA2	FISH t(11;14)	Ki-67
1	+	+	+	+	–	+	–	+	ND	–	–	ND	35%
2	+	+	+	+	–	+	–	+	+	–	–	+	80%
3	+	+	+	+	+	+	–	+	+	–	–	+	30%
4	+	+	+	+	+	+	–	–	+	–	–	+	30%
5	+	+	+	+	+	+	–	–	+	–	–	ND	30%
6	+	+	–	+	–	+	–	–	+	–	–	+	65%
7	+	+	–	+	–	+	–	–	+	–	–	+	70%
8	+	+	+	+	–	ND	–	–	+	–	–	ND	5%

+, positive; -, negative; ND, Not Done.

### Charactreistics of double-staining of EBER with CD79a/CD3 in EBV-positive MCL patients

**Figure 3:**
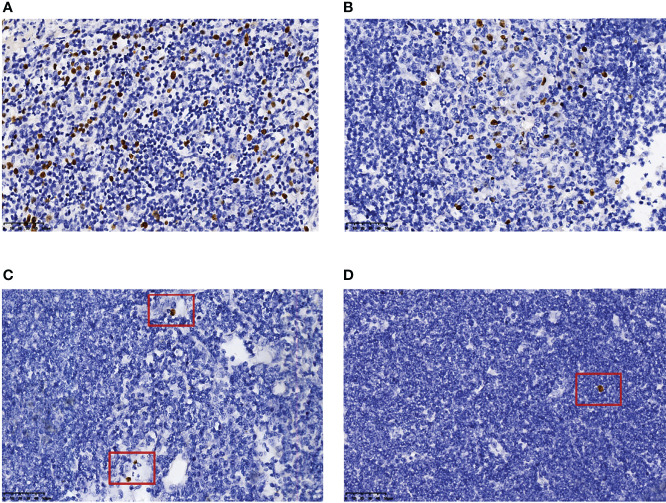
Number and distribution of EBER-positive cells. More than 100 EBER-positive cells, most of which accumulated near tumor cells **(A)** (case 1). EBER-positive cells, in the range of 10–100, distributed in the inter-tumor area **(B)** (case 4). EBER-positive cells, in the range of 1–10, scattered beside tumor cells **(C)** (case 5). One EBER-positive cell was scattered beside the tumor cells **(D)** (case 6). All images were captured at ×400 magnification.

EBER positivity was detected in eight MCL patients with EBV infection. Only one case had >100 EBER-positive cells, and most of them were distributed paratumor cells (**Figure 3A**, case 1). The number of EBER-positive cells ranged from 10 to 100 in three cases (**Figure 3B**, case 4): two cases were distributed in the inter-tumor area and one case involved scattered paratumor cells. There were three cases with 1–10 EBER-positive cells (**Figure 3C**, case 5) scattered around the tumor cells. We also observed one case with one EBER-positive cell (**Figure 3D**, case 6) scattered near the tumor cells.

**Figure 4:**
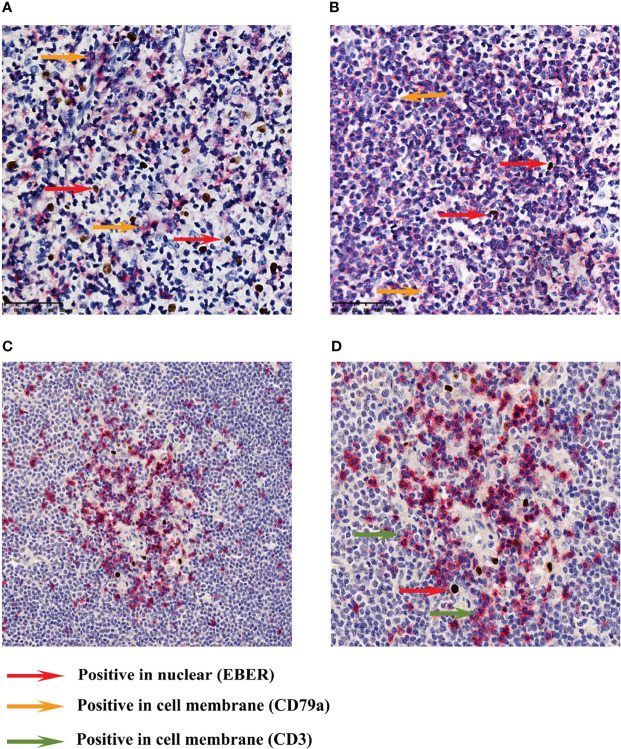
Immunohistochemical double-staining for EBER ISH and CD79a/CD3. A mixture of cell membrane-positive cells and nuclear-positive cells **(A)** (case 1). The cell membrane-positive cells were distributed in the tumor area, while most of the nuclear-positive cells were scattered in the inter-tumor area **(B)** (case 4). Most cases did not show membrane and nuclear positivity at the same time (magnification: ×400). EBER and CD3 immunohistochemical double-label staining revealed that EBER-positive cells were mainly distributed in the area of CD3-positive T cells **(C)** (case 4). A small number of EBER-positive cells could be clearly classified as CD3-positive cells **(D)** (case 4). Images **(C, D)** were captured at ×200 and ×400 magnification, respectively.

In most cells, EBER and CD79a immunohistochemical double-label staining revealed that they were not expressed on the nucleus and cell membrane of the same cell (**Figures 4A, B** cases 1 and 4). Therefore, most of the EBV-infected cells in MCL were background cells rather than tumor B cells. EBER and CD3 immunohistochemical double-label staining demonstrated that EBER-positive cells were mainly distributed in the area of CD3-positive T cells, and a small number of EBER-positive cells could be clearly classified as CD3-positive cells (**Figures 4C, D**, case 4).

## Discussion

EBV infection in MCL is rarely reported in the literature. In 2003, Tinguely et al. (11) reported a patient with chronic lymphoblastic leukemia who relapsed and was diagnosed with classical HL with MCL after 3 years of chemotherapy. EBV infection of HRS cells in the background was detected in the pathological sections of the patient. It was further found that MCL and HRS cells had the same V gene rearrangement sequence by single cell polymerase chain reaction and sequence analysis of Ig gene rearrangement. In 2003, Terasawa et al. (10) reported a case of MCL, which relapsed 8 years later and transformed into invasive diffuse large B-cell lymphoma (DLBCL); EBV was detected in the DLBCL. Southern hybridization with an EBV terminal repeat probe showed a clear monoclonal pattern in DLBCL specimens. This finding may indicate that tumor cells were infected with EBV at some point after the diagnosis of MCL. Moreover, the clonal correlation between initial MCL and subsequent DLBCL was demonstrated by IgH gene complementary determining region-III sequence analysis. In 2007, Higuchi et al. (13) reported a case of infectious mononucleosis developed into EBV-positive blastic MCL; EBV was detected in most tumor cells. In 2017, Murray et al. (14) reported a case of blastic MCL complicated with classical HL, in which EBV was detected in HRS cells and a few small lymphocytes in the background. Following fluorescence ISH, t(11;14) translocation was noted in both MCL tumor cells and HRS cells. In 2021, Kanai et al. (16) reported a patient with MCL who relapsed into nodular lymphoma with MCL and classical HL 9 years after undergoing autologous peripheral blood stem cell transplantation. EBV was detected in HRS-like cells in the MCL background, while CCND1 and SOX11 expression was attenuated or absent. However, CCND1 cleavage by fluorescence ISH showed division signals in both MCL and HRS-like cells. In addition, three patients with MCL (all with a history of mosquito bites) had EBV in the lymph nodes or markedly elevated EBV DNA in peripheral blood (9, 12, 15). Nevertheless, the incidence of EBV infection in MCL could not be assessed based on these case reports. In this study, a group of patients with MCL were screened in the pathology department of two medical institutions, and their tissues were analyzed using EBER ISH. Only EBV-positive background cells were detected in the tissues of eight patients with MCL, whereas EBV-positive cells were not detected in the remaining cases. Therefore, the total incidence of EBER positivity in MCL with background cells in this study was 5.8%. Furthermore, the cases included in this study are significantly different from previously reported EBV-positive MCL cases. The eight MCL patients with EBER positivity involved in this study did not have abnormal immune function or other lymphomas. Their histopathological manifestations were indicative of classical MCL; there was no maternal MCL or HRS-like cells in the background cells. These findings show that these eight cases of MCL with EBER positivity did not exhibit a tendency toward progression to HL or more invasive subtypes. In addition, they may indicate that EBER positivity in MCL does not necessarily occur in the disease progression stage or is not related to tumor cells infected by EBV.

MCL is an independent subtype of mature small B-cell non-HL. In recent years, it has been reported that other independent subtypes of mature small B-cell non-HL are associated with EBV infection. In 2017, Mackrides et al. (6) reported 10 cases of EBV-positive FL, which did not appear to involve immunosuppression or be complicated with other malignant tumors; EBER was expressed in the tumor cells. In 2001, Orlandi et al. (17) reported that a case of FL was transformed into invasive EBV-related DLBCL after treatment with a variety of traditional chemotherapeutic agents. In 2011, Korea Ishi et al. (18) reported a patient with FL, Kaposi’s sarcoma, and Castleman’s disease (human immunodeficiency virus negative, EBV, and human herpesvirus 8 infection). The aforementioned two patients had obvious immunosuppression. In 2018, Gong et al. (7) reported 10 cases of EBV-positive MZL. In 2013, Nassif et al. (5) reported one case of EBV-positive low-grade MZL after heart transplantation. Most patients had a history of organ transplantation, immunosuppression, and immune deficiency, and EBER positivity was detected in tumor cells. Studies have shown that transplantation, immunosuppression, and immune deficiency can induce primary infection or reactivation of EBV (19, 20). However, among the 382 cases of FL reported by Mackrides et al. (6) only six cases had scattered EBER-positive cells in the inter follicular area, which were inconsistent with the tumor cells, called “EBV positive in background cell”, and these cases were low-grade FL.

In this study, according to the morphology, immunohistochemistry, and location of the cells, most EBER-positive cells in MCL were not tumor cells but background cells. This may indicate that EBV infection often occurs in patients with FL and MZL after transplantation or immunosuppressive therapy, or with normal immune function; subsequently, the condition transforms into higher-grade or more invasive lymphoma. Of note, there was no conversion to high-grade FL or DLBCL among the six cases of low-grade FL with EBV positivity in background cells reported by Mackrides et al. (6) This suggests that, even in the presence of EBV infection, the condition may not necessarily develop into a higher grade or more invasive lymphoma. This may be related to the fact that EBV does not infect tumor cells or the time of infection. According to Camacho Casta ñ EDA FI (8), one case of intranodal MZL was completely resolved after six cycles of chemotherapy. Lymph node biopsy showed scattered EBV-positive cells; 10 years later, the condition transformed into EBV-positive DLBCL. However, the article did not clearly state that the EBV-positive cells in the patient’s first biopsy were tumor cells. In 2017, Ohashi et al. (21) found 29 patients with EBV-negative DLBCL and EBV-positive background cells among 664 patients with DLBCL. Their clinical manifestations were not significantly different from those of patients with EBV-negative DLBCL without EBV-positive background cells; nevertheless, their prognosis was poor. Among them, 33% (7/21) of patients expired within 2 years after diagnosis, and the patients with the longest survival time were followed up for 76 months, confirming the poor prognosis of these patients. This suggests that infection of background cells with EBV is a long early pathogenic process versus infection of tumor cells with this virus. In 1995, Hummel et al. (22) proposed that in case of EBV infection of a small number of tumors and paratumoral cells, the infection may occur after malignant transformation of the tumor. In the eight cases examined in this study, most EBV-infected cells were scattered near the tumor cells, EBER-positive cells gathered near the tumor cells in only one case, and EBER-positive cells were few. There was only one case with >100 cells, most cases had 10–100 cells, and one case had only one EBER-positive cell. Therefore, we hypothesized that infection of MCL patients with EBV may occur after the diagnosis of MCL.

We found that the immunophenotype of MCL with EBER positivity was similar to that of MCL without EBER positivity. Generally, patients with classical MCL express B cell markers, CD5, CCND1, and SOX11, and most exhibit t(11;14) chromosome rearrangement and translocation. Although the eight cases of MCL with EBV infection in this study demonstrated heterogeneous immunohistochemical expression, some cases did not express CD5 and SOX11, while other showed abnormal expression of BCL-6. However, these heterogeneities did not reveal statistically significant differences versus cases of MCL without EBV infection. In this study, eight cases expressed CD20, CD79a, and CCND1; of those, six cases expressed CD5. Some studies have found that patients with CD5-positive and CD5-negative MCL exhibit similar clinicopathological features to a large extent; however, patients with CD5-negative MCL have a better prognosis than those with CD5-positive MCL (23). Of the eight EBV-positive cases, five did not express SOX11, whereas three did. Studies have shown that SOX11-negative MCL is linked to a higher incidence of leukemia without lymph node involvement, classic histological morphology, and a lower Ki-67 index (24). In contrast, the clinical course of leukemic MCL without lymph node involvement is less aggressive than that of classical MCL. The BCL-6 gene encodes a transcriptional repressor protein that is mainly confined to germinal center B cells, while MCL is derived from pre-germinal center B cells or mantle B cells. In this study, five cases did not express BCL-6, whereas three cases did. Although abnormal BCL-6 expression has been rarely reported in MCL, it is associated with a high Ki-67 index and cytogenetic aberrations involving the BCL-6 gene (25, 26). LMP1 and EBNA2 were not detected in the eight cases, indicating that they were not in the latent infection mode II or III (27). This finding was consistent with the results reported by Hummel et al. (28) in 1992.

The clinical features of the eight MCL patients with EBER positivity included in this study were similar to those of MCL patients without EBV infection. There were no significant differences in the age of onset, sex ratio, and most clinical manifestations. However, MCL patients with EBER positivity had elevated LDH, progressive anemia, and MIPI groups versus those without EBV infection. Most patients in this study were males and aged ≥60 years. Furthermore, most patients were in the late clinical stage (Ann Arbor stage III or IV), with multiple lymph node enlargement and anemia. Extranodal lesions were common (gastrointestinal tract and parotid gland). Approximately half of the patients had hepatosplenomegaly and bone marrow involvement; however, there were no B symptoms. Most patients had elevated LDH, and a few patients had elevated absolute values of β2-MG and monocytes. The increase of the first two indices indicated a poor prognosis (29, 30). In 2020, Zhou et al. (31) showed more EBV DNA-positive patients versus EBV DNA-negative patients in the group with high absolute monocytes. Ki-67 ≥30% in MCL is unfavorable to the overall survival of patients (32). Although Ki-67 was ≥30% in half of the patients analyzed in this study, the rate was similar to that observed in MCL patients without EBV infection. Follow-up analysis revealed that the 5-year overall survival rate of patients in the low-risk group was 60%, and the median survival time of patients in the medium-risk group was 51 months. The prognosis of patients in the high-risk group was poor, and the median survival time was only 29 months. Only six patients were evaluated for MIPI in this study, because two patients were not tested for LDH. All six cases were in the medium- and high-risk groups (four and two cases, respectively), suggesting that the prognosis of these patients may be slightly worse than that of other patients. Based on the follow-up of this study, the overall survival time of four patients was as short as 6 months and as long as 5 years.

**Figure 5:**
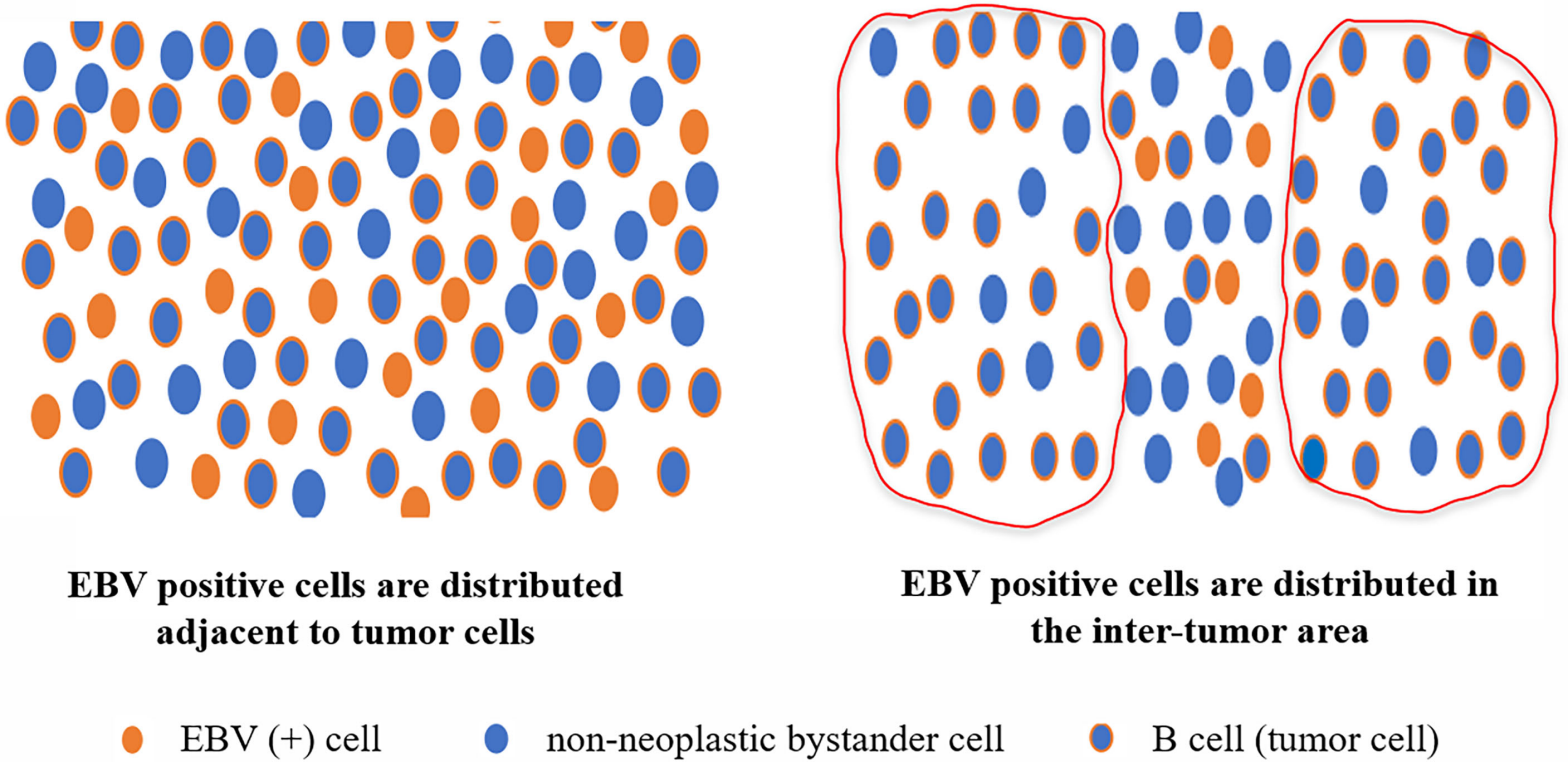
Distribution pattern of EBER-positive cells in MCL with EBER positivity. According to their morphology, immunohistochemistry, and location, most EBER-positive cells in MCL were not tumor cells, and most of the EBV-infected cells were background cells in MCL.

In summary, the incidence of MCL with EBER positivity was low and mostly in background cells, in contrast to other mature small B-cell non-HL with EBER positivity (**Figure 5**). In pathological diagnosis, pathomorphology, immunohistochemical staining, and clinical features cannot be used to evaluate the presence of EBV infection in MCL. This is because the clinicopathological features of MCL with EBV infection are similar to those of MCL without EBV infection. Patients with elevated LDH, progressive anemia status, and medium- or high-risk MIPI grouping are more likely to have EBV infection. Pathologists can detect EBV in patient tissue to determine the prognosis and disease progression. Additional research studies involving more samples and data are warranted to further clarify the clinicopathological features and biological significance of MCL with EBV infection.

## Data availability statement

The raw data supporting the conclusions of this article will be made available by the authors, without undue reservation.

## Ethics statement

Studies were performed according to the Declaration of Helsinki. The procedures were reviewed and approved by the Ethics Committee of Chongqing University Cancer Hospital (CZLS2022065-A). The consent for publication was obtained from all participants for the publication of this study.

## Author contributions

YL, HZ, XJL and FLZ designed the experiments; XJL, FLZ, SJL, JNF, XL, YYW, TW, YP, YIP, JBX, JL, CQH and JZ performed the experiments; YDJ, FLZ, WYJ, YXZ, SKL, FL, XJL and YL helped in data analysis and paper writing. All authors contributed to the article and approved the submitted version

## Funding

This work was supported by the Performance Incentive Guidance for Scientific Research Institution of Chongqing (No. cstc2020jxjl130013) and the Fundamental Research Funds for the Central University (No. 2019CDYGZD002).

## Conflict of interest

The authors declare that the research was conducted in the absence of any commercial or financial relationships that could be construed as a potential conflict of interest.

## Publisher’s note

All claims expressed in this article are solely those of the authors and do not necessarily represent those of their affiliated organizations, or those of the publisher, the editors and the reviewers. Any product that may be evaluated in this article, or claim that may be made by its manufacturer, is not guaranteed or endorsed by the publisher.
